# Circadian Effects on Performance and Effort in Collegiate Swimmers

**DOI:** 10.5334/jcr.165

**Published:** 2018-08-03

**Authors:** Austin Anderson, Gillian Murray, Meaghan Herlihy, Chloe Weiss, Jacob King, Ellen Hutchinson, Neil Albert, Krista K. Ingram

**Affiliations:** 1 Department of Biology, Colgate University, NY, US; 2 Department of Psychology, Colgate University, NY, US

**Keywords:** diurnal preference, chronotype, circadian phenotype, circadian genotype, PER3, athletic performance, physiological effort, alpha amylase

## Abstract

Although individual athletic performance generally tends to peak in the evening, individuals who exhibit a strong diurnal preference perform better closer to their circadian peak. Time-of-day performance effects are influenced by circadian phenotype (diurnal preference and chronotype—sleep-wake patterns), homeostatic energy reserves and, potentially, genotype, yet little is known about how these factors influence physiological effort. Here, we investigate the effects of time of day, diurnal preference, chronotype, and *PER3* (a circadian clock gene) genotype on both effort and performance in a population of Division I collegiate swimmers (n = 27). Participants competed in 200m time trials at 7:00 and 19:00 and were sampled pre- and post-trial for salivary α-amylase levels (as a measure of physiological effort), allowing for per-individual measures of performance and physiological effort. Hair samples were collected for genotype analysis (a variable-number tandem-repeat (VNTR) and a single nucleotide polymorphism (SNP) in *PER3*). Our results indicate significant and parallel time-of-day by circadian phenotype effects on swim performance and effort; evening-type swimmers swam on average 6% slower with 50% greater α-amylase levels in the morning than they did in the evening, and morning types required 5–7 times more effort in the evening trial to achieve the same performance result as the morning trial. In addition, our results suggest that these performance effects may be influenced by gene (circadian clock gene *PER3* variants) by environment (time of day) interactions. Participants homozygous for the *PER3*^4,4^ length variant (rs57875989) or who possess a single G-allele at *PER3* SNP rs228697 swam 3–6% slower in the morning. Overall, these results suggest that intra-individual variation in athletic performance and effort with time of day is associated with circadian phenotype and *PER3* genotype.

## Introduction

Athletic performance generally peaks in the afternoon when physiological processes fueling metabolic activity are at their peak [36,41,9]. This effect can be seen at both the individual and team levels, e.g. professional American football teams on the west coast of the United States gain a significant athletic advantage over travelling east coast teams during nighttime games [33] and team and individual performance suffer during daytime games following eastward travel because of the lag in reaching afternoon peak potential [29,44,7,34].

At the individual level, peak performance is also influenced by circadian phenotypes, including an individual’s diurnal preference for morning or evening activity patterns [5,16] and chronotype, an individual’s sleep-wake phenotype. Athletes with extreme morning or evening phenotypes tend to perform better near their circadian peak in endurance activities [5,12,28,26], as well as in strength training [6,13,32,10,21]. Interestingly, these diurnal effects may be strong enough to shape the distribution of circadian phenotypes in particular endurance sports that regularly compete in the morning; morning-types are more prevalent among elite runners, cyclists, and triathletes [23,20].

In addition to performance, chronotype influences time of day differences in psychophysiological responses to exertion, including rate of perceived exertion (RPE), heart rate (HRV) and mood [38,19,28,31,39] reviewed in [40]. Although peak athletic performance generally coincides with peak body temperature in early evening, daily peaks in body temperature, melatonin and serum cortisol differ between morning and evening chronotypes [1,2]. Thus, intra-individual differences in internal physiology are likely to impact peak performance times of extreme chronotypes. However, most studies to date have focused on self-reported RPE or fatigue scores as measures of physiological effort, with relatively few studies including biological measures of physical exertion (i.e. hormones such as cortisol or salivary alpha-amylase) (but see [4,39,3]).

Here, we test the concept of a ‘morning handicap’ in evening-types, who are expected to struggle in morning competitions because their circadian timing and physiological activity both peak in the afternoon; conversely, circadian timing in morning types is asynchronous with peak afternoon physiological activity, resulting in less variability in performance across the day. Furthermore, we investigate whether molecular markers of circadian genotype and physiological markers of stress provide robust measures of the influence of chronotype and energetic effort on time of day athletic performance in Division I collegiate swimmers.

To compare performance of self-reported circadian phenotypes, we first quantified diurnal preference with the commonly used Horne-Östberg Morning-Eveningness Questionnaire (MEQ) [15] and measured the sleep-wake chronotype of individuals using the mid-sleep on free days corrected for sleep debt from work days (MSFsc) from the Munich Chronotype Questionnaire. Morning-type individuals (MT) are characterized by early wake times and peak-alertness during the mid-morning, while evening-types (ET) are characterized by delayed wake times and peak-alertness during the late afternoon or evening. We test the hypothesis that evening-types show greater variance in time-of-day performance than morning-types, with poorer performance expected in the morning trial period for ET.

Because individuals may compensate for performance variability by working harder at non-peak times, we measured how effort expended during the time trials changes with time of day using self-report surveys and assays of salivary α-amylase, a non-invasive, biological marker of physiological stress synchronous with the production of plasma catecholamines. We test the hypothesis that salivary α-amylase levels will be higher during testing in the non-circadian peak times for individuals (i.e. higher in the morning trials in evening-types).

Time-of-day performance variation may also be influenced by an individual’s genotype and, in particular, by allele variants in circadian clock genes that are associated with diurnal preference, like PER3. In this study, we investigated whether the presence of a *PER3*-G allele or a *PER3^4/4^* genotype, genotypes that are associated with eveningness, had an effect on athletic performance. We test the hypothesis that individuals with a *PER3*-G allele and/or a *PER3^4/4^* genotype experience a handicap in performance and physiological effort in the morning.

## Materials and Methods

### Subjects

Twenty-seven individuals from the Colgate University varsity swimming and diving team voluntarily participated in this study: 8 males (average weight, 78 kg, and height range, 1.75–1.93 m) and 19 females (average weight, 62 kg, and height range 1.55–1.83 m) with an age range from 18–22 years. Informed consent was obtained from the participants before the sampling was conducted. All methods were developed in agreement with the Declaration of Helsinki; procedures and consent forms were approved by the Institutional Review Board at Colgate University (FR-F16-17a and FR-F16-17b).

### Experimental Design

#### Participant Sampling

All participants completed a 200m freestyle time trial at two sessions: one morning (7:00) and one evening (19:00) session. Individuals provided three saliva samples per session: baseline, pre- and post-task saliva samples. During their first session, participants also completed the Horne-Östberg Morningness-Eveningness Questionnaire (MEQ)[15] and provided hair samples for genetic analysis. Morning and evening sessions were randomized across individuals to minimize order effects.

#### Time Trial

Following a 10-minute warm-up swim period, participants swam a timed 200-meter freestyle trial in Colgate University’s varsity swimming pool (50-meter length). All participants started the trial in the water with a wall push-off, used tumble-turns between laps, and swam alone. Swimmers then completed the athletic task again during the second session (7:00 or 19:00). Each session was separated by 12 h or 24 h to accommodate varsity-training schedules. We calculated *per-individual* percent differences in morning versus evening performance relative to morning values (the ‘morning handicap’ = (M-E)/E) following 200m time trials.

### Circadian Behavioral Phenotype

The Horne-Östberg Morningness-Eveningness Questionnaire (MEQ) was administered to each participant to determine self-reported diurnal preference. The survey consists of nineteen questions assessing diurnal preference by analyzing subject time of day preferences for certain activities (sleeping, alertness, etc.). Individuals with high scores (>59) represent a morning lark chronotype and individuals with low scores (<41) represent an evening owl chronotype. Out of 27 swimmers, 3 self-reported as morning types (MEQ >59) and 7 self-reported as evening-types (MEQ <41). To equilibrate sample sizes, we analyzed the top and bottom MEQ quartiles (seven individuals with highest (morning-type or MT) and lowest (evening-type or ET) MEQ scores). We calculated the percent difference in morning-evening performance (relative to morning values) following 200m time trials between MT and ET groups.

We measured a participant’s sleep-wake chronotype using the mid-sleep on free days value corrected for sleep debt accumulated during the week (MSFsc) from the Munich Chronotype Questionnaire (n = 13, MT(<5:00) = 4, ET(>6:00 = 4), NT = 5; MSFsc cutoffs were based on a larger sample from this undergraduate population). We used individuals with the lowest (morning-type or MT) and highest (evening-type or ET) MSFsc scores to calculate the percent difference in morning-evening performance (relative to morning values) following 200m time trials between these two groups. Circadian phenotypes estimated by MEQ and MSFsc methods were significantly correlated (Pearson’s r = –0.67, p = 0.011).

Sleepiness is known to influence metabolic physiology and may modulate time of day performance and effort independent of circadian effects. Before the time trials, athletes completed the short form PROMIS sleep disturbance questionnaire [45] allowing us to compare performance results with a standardized measure of sleep disturbance. T-scores generated from this survey represent a standardized score with a mean of 50 and SD of 10. Higher T-scores indicate poor sleep with values greater than 55 and 60 representing mild and severe sleep disturbance, respectively. We compared the time trial performance of swimmers from the lower and higher quartiles of the sleep disturbance results.

### α-Amylase Analysis

Salivary α-amylase was analyzed as a measure of physiological effort. Salivary α-amylase production is synchronous with the production of plasma catecholamines, particularly norepinephrine, and therefore provides a non-invasive, biological marker of physiological stress [27,18,17,8]. Participants provided one saliva sample on-site each time they arrived, for a baseline measure, and a second sample immediately after the swim trial. Samples were analyzed following the Salimetrics salivary α-amylase kinetic enzyme assay protocol. We calculated *per-individual* changes in salivary α-amylase post-exercise in morning versus evening sessions after normalizing to pre-exercise levels. Perceived effort was measured with two survey questions rated on a 10-point scale—How challenging was the time trial and How tired do you feel following the time trial? We calculated *per-individual* changes in perceived effort scores.

### Genotype Analysis

Twenty strands of hair were collected from each participant to characterize their genotype for *PER3* SNP rs228697 and *PER3* VNTR rs57875989. Following digestion of hair at 37°C for 24 hours, DNA was extracted and purified with the Qiagen DNAeasy Micro Kit. Genotyping for PER3 SNP rs228697 was performed using a TaqMan SNP Genotyping assay (Applied Biosystems, Foster City, CA) on an ABI 3700HT real-time qPCR instrument. Participants were identified as homozygous or heterozygous for the major allele C or the minor allele G.

To measure the VNTR length polymorphism of 54 base pairs (bp) in exon 18 of the PER3 gene, we used a fragment length analysis on an ABI 3100 sequencer. The following PCR primers were used with the forward primer fluorescently labeled with 6-FAM: forward, 5′-CAAAATTTTATGACACTACCAGAATGGCTGAC-3′, and reverse, 5′-AA CCTTGTACTTCCACATCAGTGCCTGG-3′ [11]. The PCR was performed in a 25-uL volume using Qiagen PCR Mastermix. Positive and negative DNA controls were included with each PCR plate of samples. The PCR cycling conditions were 3 min. at 94°C, followed by 35 cycles of 45 sec. at 94°C, 45 sec. at 58°C, and 45 sec. at 72°C, with a final step at 72°C for 3 min. PER3 alleles were separated by capillary electrophoresis and sized using ABI ROX standards. Participants were identified as *PER3^4/4^, PER3^4/5^* or *PER3^5/5^*.

### Statistical Analyses

Differences in swim times and morning handicap values between diurnal preference types, chronotypes, genotypes and sleep phenotypes were tested with one-way ANOVAs and/or t-tests. Differences in morning versus evening α-amylase levels and in perceived effort responses post-exercise (M-E) between diurnal preference types and chronotypes were tested with paired t-tests. Odds ratio tests or contingency tables were performed to test for allele frequencies differences by *PER3* chronotype. Genotypes (CC versus CG/GG and *PER3^4/4^* versus *PER3^4/5^*) were compared by average MEQ score using t-tests. To investigate the association effect of multiple *PER3* mutations, we compared individuals with the genetic variants for eveningness (G allele and *PER3*^4/4^ genotype, the PER3 haplotype) against all other genotypes with t-tests on average MEQ score and time-of-day athletic performance.

## Results

### Significant ‘morning handicap’ for evening-type athletes

**Figure 1:**
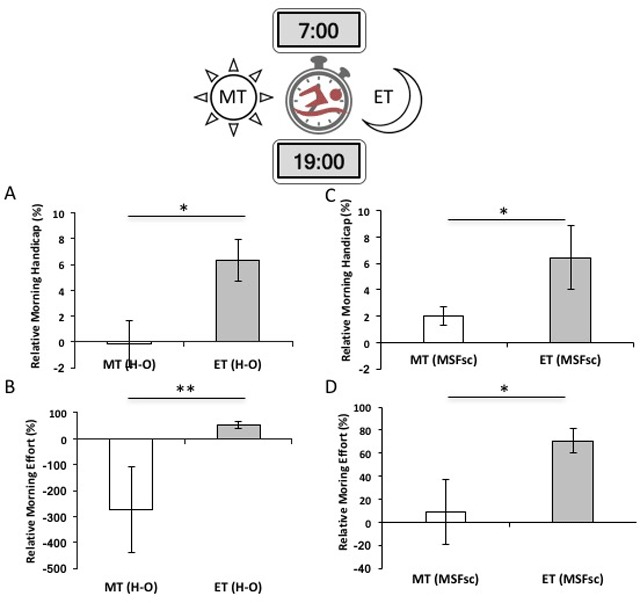
Relative morning handicap representing the average per-individual time-of-day effects on athletic performance and effort in self-reported circadian chronotypes (±SE). **A** & **B)** Morning handicap on performance is measured as the average per-individual time trial score ((AM–PM)/PM). Individual trial time (in seconds) differences (AM–PM) are normalized to evening times; values shown represent relative percent differences in performance. A) Evening-types, as measured by diurnal preference (HO-MEQ), have a significantly greater morning handicap than morning-types, swimming 6% slower in morning (07h00) than evening (19h00) 200-meter timed-trials (t = 2.70, df = 12, p = 0.017). B) Morning handicap on performance in chronotypes (MSFsc). Differences between ET and MT are significant (t = 2.45, df = 11, p = 0.034). **C** & **D)** Time-of-day effects on physiological effort measured by salivary α-amylase levels. Data are reported as average difference in α-amylase concentration between the morning and evening trials (relative to individual- and time-specific baselines; ±SE). C) For circadian phenotypes measured by diurnal preference (HO-MEQ), both MT and ET participants exert more effort at off-peak times and there is a significant relative percent difference in diurnal effort between MT and ET (t = 2.64, df = 10, p = 0.014). D) For chronotypes (MSFsc), only evening-types exert more effort at their non-peak morning trials and differences between MT and ET are marginally significant (t = 2.64, df = 10, p = 0.05).

Overall, participants were, on average, 2.5 ± 0.88% (SE) slower in the morning, with an average of 3.3 ± 1.26 seconds added during morning time trials. Individuals who report an evening preference (H-O MEQ ET) were 6.3% slower, on average, in the morning time trial (split difference (AM–PM) for diurnal preference: *M*_ET(H-O)_ = 8.7 ± 0.02 seconds; *M*_MT(H-O)_ = –2 ± 2.46 seconds. Evening- chronotypes (MSFsc ET) were 3.2% slower, on average, in the morning trial (split difference (AM–PM) for chronotype: *M*_ET(MSFsc)_ = 6.4 ± 2.43 seconds; *M*_MT(MSFsc)_ = 2.0 ± 0.67 seconds). The relative morning speed of ET was significantly different from MT for both diurnal preference (t = 2.70, df = 12, p = 0.017) and chronotype (t = 2.45, df = 11, p = 0.034; Figure 1a and c), suggesting a significant morning handicap for self-reported evening-types.

### Extreme circadian phenotypes exert significantly more physiological effort at non-peak times

Both MT and ET showed significant post-exercise increases in α-amylase levels above baseline at non-peak times relative to peak times; for diurnal preference, α-amylase levels rose 50% in ET in the morning sessions and greater than 250% in MT in the evening sessions (*M*_ET(MSFsc)_ = 70.78% ± 10.42, *M*_MT(MSFsc)_ = 8.9% ± 28.43, t = 2.64, df = 10, p = 0.014; Figure 1b). For the chronotype measure, α-amylase levels rose 71% in ET in the morning sessions but did not differ for MT in the evening sessions (*M*_ET(H-O)_ = 51.96% ± 12.07, *M*_MT(H-O)_ = –274.35% ± 164.00, t = 2.64, df = 10, p = 0.05; Figure 1d).

Interestingly, athletes reported no significant differences in perceived effort at peak versus non-peak times (MT—*effort*: t = 1.59, df = 6, p = 0.187; ET—*challenge*: t = 0.70, df = 7, p = 0.50; ET—*effort*: t = 1.12, df = 7, p = 0.283). The incongruity between the physiological and self-reported effort data suggests that these self-reported measures of perceived effort did not accurately reflect physiological energy expenditure.

Athletes did vary in levels of sleep disturbance ranging from raw scores of 13–28 on the sleep scale, indicating sleep disturbance T-scores of between 41.4 and 58.3. We found no difference in morning performance between individuals with higher or lower sleep disturbance (t = 0.94, df = 12; p = 0.367; M_low_ = 2.44 ± 1.66, M_high_ = 1.38 ± 2.41). Although sleep disturbance was significantly inversely correlated with MEQ scores, with lower MEQ scores (evening-types) reporting higher sleep disturbance (r = 0.55, p = 0.004), sleep quality did not appear to influence time of day performance.

### Time of day performance changes associated with PER3 genotype

**Figure 2:**
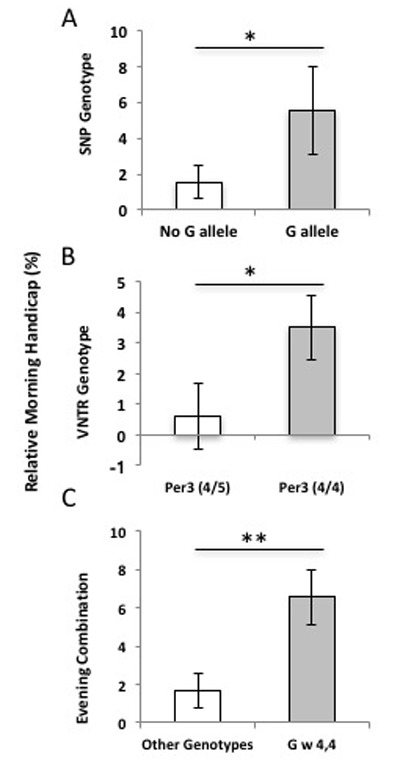
Influence of the *PER3* SNP and VNTR polymorphisms on athletic performance. Relative morning handicap represents the average per-individual time-of-day effects (M-E/E) on athletic performance in *PER3* genotypes (±SE). **A)** Participants with a G allele are significantly slower in the morning swim trial (*M*_w/G_ = 8.64 ± 0.024) than those homozygous for the C allele (*M*_CC_ = 1.847s ± 1.140) representing an 6% decrease in morning performance in individuals with G-allele (t = 2.313, df = 23, p = 0.015). **B)** *PER3^4/4^* genotypes show a 3–4% slower trend in 200m swim performance, but this trend is not significant (t = 1.685, df = 23, p = 0.053). Participants with *PER3^4/4^* genotypes tend to swim nearly 5 seconds slower in the morning trials (*M*_4/4_ = 4.830 ± 1.52) compared to participants with *PER3^4/5^* genotypes (*M*_4/5_ = 0.667 ± 2.116). **C)** Participants with the PER3 haplotype swam 10 seconds (*M*_eve_ = 10.325 ± 2.087) slower in the morning, on average, while all other genotype combinations swim only 2 seconds slower (*M*_other_ = 2.000 ± 1.129; t = 2.905, df = 24, p = 0.008), this represents an 6.5 % slower individual morning performance in athletes with both evening-type polymorphisms (t = 7.26, df = 24, p = 0.013).

**Figure 3:**
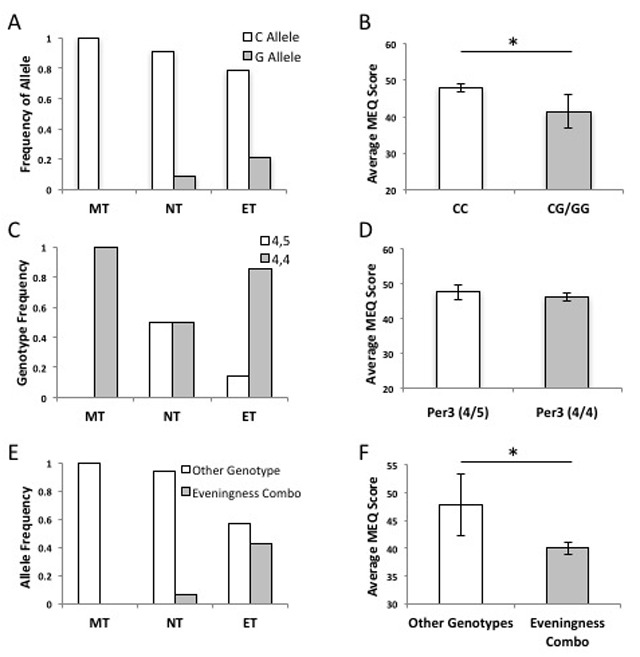
Allele and chronotype frequency and chronotype association of the *PER3* SNP and VNTR in elite swimmers. **A)** The *PER3* G allele is more frequent in ET (n = 7) than in MT and NT (n = 19) (OR = 6.800, phi = 1.45, p = 0.148). **B)** Genotypes with a G present have a significantly lower average MEQ score (*M* = 41.4 ± 4.55) than CC genotypes (*M* = 47.9 ± 1.11) (t = 13.7, df = 24, p = 0.046). **C)** The *PER3* 5 repeat allele is most prevalent in intermediates but does not have a significant association with a specific chronotype (OR = 0.208, phi = 0.28, p = 0.208). **D)** Participants with a heterozygous VNTR genotype (*PER3^4/5^*) do not have higher average MEQ scores (*M* = 47.6 ± 3.47) than participants with the homozygous (*PER3^4/4^*) genotype (*M* = 46 ± 8.10; t = 0.545, df = 23, p = 0.591). **E)** The PER3 ‘evening’ haplotype (a G-allele and *PER3^4/4^*) is more frequent in ET than in MT and NT (OR = 8.000, phi = 1.63, p = 0.103). **F)** The average MEQ score is significantly lower in participants with the PER3 eveningness haplotype—individuals homozygous for the 4-repeat VNTR (*PER3^4/4^* genotype) and possessing a G allele—than the average of all the other genotypes (M_eve_ = 40 ± 5.583; M_other_ = 47.86 ± 1.082; t = 5.64, df = 23, p = 0.026).

The *PER3* SNP G allele tended to be more prevalent in individuals with evening preference, but this trend was not significant (OR = 0.156, p = 0.101; Figure 3A). No morning-types had the G allele. Heterozygotes (CG) and homozygotes for the minor allele (GG) had significantly lower average MEQ scores than CC homozygotes (t = 13.7, df = 24, p = 0.046; Figure 3B) and the average MEQ score for the CG/GG genotype was 41.4 ± 4.55, which includes values classified as evening diurnal preference on the HO scale. Participants with the CG *PER3* genotype had faster 200-meter swim times in the evening session than participants homozygous for the C allele (t = 2.313, df = 24, p = 0.015), representing a 6% increase in split times for evening-types who swam in the morning (Figure 2A). Participants with the G allele (CG or GG) swam, on average, nearly 9 seconds slower in the morning (*M*_w/G_ = 8.64 ± 0.024), while individuals with the CC genotype swam, on average, only 2 seconds slower in the morning (*M*_CC_ = 1.847s ± 1.140; t = 2.67, df = 24, p = 0.0137).

The PER3 VNTR polymorphism was not strongly associated with diurnal preference (OR = 0.208, phi = 0.28, p = 0.208; Figure 3C) and we sampled no *PER3^5/5^* homozygotes. Furthermore, we found no significant difference in average MEQ scores between participants with the *PER3^4/5^* and *PER3^4/4^* genotypes (t = 0.545, df = 23, p = 0.591; Figure 3D). The *PER3* VNTR genotype did not have a significant effect on time of day athletic performance (t = 1.685, df = 23, p = 0.053; Figure 2B); we measured only a 3–4% slower trend in 200m swim performance in *PER3^4/4^* genotypes (M_4/4_ = 4.830 ± 1.52; M_4/5_ = 0.667 ± 2.116; t = 1.98, df = 23, p = 0.059).

We also tested whether the PER3 haplotype (including the SNP_G allele and VNTR 4,4) conferred a stronger effect than single polymorphisms. The average MEQ score was significantly lower in participants with the PER3 haplotype—individuals homozygous for the 4-repeat VNTR (*PER3^4/4^* genotype) and possessing a G allele (*M*_eve_ = 40 ± 5.583; *M*_other_ = 47.86 ± 1.082; t = 5.64, df = 23, p = 0.026; Figure 3F). This haplotype also had a significant effect on athletic performance; participants with the two PER3 polymorphisms swam 10 seconds (M_eve_ = 10.325 ± 2.087) slower in the morning, on average, while all other genotype combinations swam only 2 seconds slower (M_other_ = 2.000 ± 1.129; t = 2.905, df = 23, p = 0.008), this represents a 6.5% slower individual morning performance in athletes with the PER3 haplotype (t = 7.26, df = 23, p = 0.013; Figure 2C).

## Discussion

Our results suggest that time-of-day effects on athletic performance and effort are influenced by an individual’s circadian behavioral phenotype and are associated with molecular and physiological differences. Elite swimmers who self-report as evening-types swim up to 6% slower and expend 50–70% more effort in the morning. Our results show a stronger effect on non-circadian peak performance and effort in evening versus morning chronotypes, as expected, due to synchrony of peak circadian activity with peak afternoon overall physiological performance in evening-types. If circadian diurnal preference influences performance, the morning handicap should be amplified in evening-types, who experience both a physiological and circadian peak in the evening relative to morning trials, and this expectation is supported by our data.

Interestingly, these performance differences can also be predicted by *PER3* genotype; individuals with inherited polymorphisms in a gene previously associated with diurnal preference perform better in evening trials, independent of self-reported circadian phenotype. The two specific polymorphisms examined in this study alter coding regions containing phosphorylation sites. Reduced phosphorylation of *PER3* may be associated with a lengthening of period and a delay in the circadian phase, both of which are typically associated with an evening phenotype [14,46,25,24]. This delay in circadian phase exacerbates the social jetlag (or misalignment of internal rhythms with daily sleep patterns) [42,30,43], and likely enhances the offset in the physiological peak of evening-types. Consequently, evening-types with these genotypes can be expected to perform better even later in the afternoon—and even worse in the morning. Since the combined effect of both polymorphisms on performance is only slightly larger than the effect of the *PER3* SNP alone, it appears that there are few or no additive effects for this haplotype.

Time-of-day performance effects have been largely investigated in highly trained athletes because of the implications on competitive success in demanding environments. Such studies commonly use self-report diurnal preference methods to understand the influence of both the circadian clock and an individual’s limited energy reserves on physical performance. Performance differences suggest that environmentally- or socially-induced factors (i.e. acute lack of sleep) may affect diurnal physical performance, and likely amplify the effects of intrinsic circadian misalignment on time-of-day performance.

In contrast, we found that self-report measures of effort may drastically underestimate time-of-day effects on physiological stress. We found no association between perceived effort and physiological effort as measured by salivary α-amylase. Because swimmers in this study regularly trained in both morning and evening sessions during pre-season, our results do not support the finding that habitual training at a particular time of day reduces physiological effort [28], evening-type swimmers experienced higher physiological stress in morning time trials than in evening time trials and vice versa. It is possible that habitual training at non-circadian peak time minimizes the effort handicap, but it does not appear to eliminate it. Because one can now disassociate effortful training from optimal performance training using physiological measures, like salivary a-amylase, further studies could test whether habitual training under high effort, non-peak times improves performance more than training at peak circadian times. Future studies could also consider the alternative—whether habitual training at peak times, when physiological systems are working optimally [35], achieves higher performance gains.

The results of our study suggest that physical activity during local minima of the circadian cycle leads to impaired performance and dramatically increased physiological stress. Thus, our findings have implications for athletic communities in terms of practice/workout schedules to enhance athletic potential—in particular, a ‘one time fits all’ schedule might not optimize performance for all athletes. Coaches and trainers may want to adopt diverse or flexible schedules, and it should be noted that the optimal scheduling times might differ for strength versus speed versus endurance training [21,6,22].

Aside from effects on peak performance, the misalignment of the internal molecular clock with metabolic and other physiological systems impacting bodily responses to stress and fatigue may impact recovery time [37] and individual health. A critical concern for athletes is avoiding potential injuries that may result from circadian misalignment with today’s demanding schedules. For collegiate athletes, like the participants in this study, the combined energetic demands of performing well on both cognitive and physical dimensions on a daily basis may warrant an even greater need to optimize scheduling to minimize fatigue and bodily or mental stress.

The limitations of this study include the use of a non-validated exertion scale, a relatively small sample size for extreme morning- and evening-types (and the subsequent use of quartiles to analyze MEQ scores), and a focus on speed performance in one sport only. Future studies should explore the effects of individual time-of-day variance in all aspects of athletic training and performance to better understand the effects of circadian phenotype on recovery, exertion and athlete health. Recognizing the limitations of physiological performance and effort will help ensure that we have the tools to tailor training and competition environments to the individual.
